# A chloroplast-targeted DnaJ protein contributes to maintenance of photosystem II under chilling stress

**DOI:** 10.1093/jxb/ert357

**Published:** 2013-11-13

**Authors:** Fanying Kong, Yongsheng Deng, Bin Zhou, Guodong Wang, Yu Wang, Qingwei Meng

**Affiliations:** College of Life Science, State Key Laboratory of Crop Biology, Shandong Agricultural University, Tai’an, Shandong 271018, PR China

**Keywords:** Chilling stress, D1 protein, DnaJ protein, Hsp70, photosystem II, tomato.

## Abstract

DnaJ proteins act as essential molecular chaperones in protein homeostasis and protein complex stabilization under stress conditions. The roles of a tomato (*Lycopersicon esculentum*) *c*hloroplast-targeted *D*na*J* protein (LeCDJ1), whose expression was upregulated by treatment at 4 and 42 °C, and with high light, NaCl, polyethylene glycol, and H_2_O_2_, were investigated here using sense and antisense transgenic tomatoes. The sense plants exhibited not only higher chlorophyll content, fresh weight and net photosynthetic rate, but also lower accumulation of reactive oxygen species and membrane damage under chilling stress. Moreover, the maximal photochemistry efficiency of photosystem II (PSII) (*F*_v_/*F*_m_) and D1 protein content were higher in the sense plants and lower in the antisense plants, and the photoinhibitory quenching was lower in the sense plants and higher in the antisense plants, suggesting that the inhibition of PSII was less severe in the sense plants and more severe in the antisense plants compared with the wild type. Furthermore, the PSII protein complexes were also more stable in the sense plants. Interestingly, the sense plants treated with streptomycin (SM), an inhibitor of organellar translation, still showed higher *F*_v_/*F*_m_, D1 protein content and PSII stability than the SM-untreated antisense plants. This finding suggested that the protective effect of LeCDJ1 on PSII was, at least partially, independent of D1 protein synthesis. Furthermore, chloroplast heat-shock protein 70 was identified as the partner of LeCDJ1. These results indicate that LeCDJ1 has essential functions in maintaining PSII under chilling stress.

## Abstract (precis)

A novel tomato chloroplast-targeted DnaJ protein, LeCDJ1 was found to contribute to the maintenance of photosystem II under chilling stress and this maintenance effect was, at least partially, independent of D1 protein synthesis

## Introduction

Photosystem II (PSII) is a large pigment–protein complex in the thylakoid membrane that performs the key reactions of photosynthesis (Shi *et al.*, 2012). It exists mainly in dimeric form with the monomer containing at least 27–28 subunits (Rochaix, 2011). A distinct feature of PSII is that it is particularly prone to photo-oxidative damage under abiotic stresses (Aro *et al.*, 1993). Chilling stress is one of the most significant environmental stresses on agricultural plants. Numerous studies have shown that chilling stress inhibits PSII activity (Li *et al.*, 2010; Duan *et al.*, 2012). Plants exposed to chilling stress show reduced metabolic rates (Partelli *et al.*, 2009). This often leads to photoinhibition, which is referred to as inhibition of the activity of PSII, and is due to an imbalance between the rate of PSII damage and repair. The main target of PSII damage is the PSII reaction centre D1 protein, and the damaged D1 protein must be repaired by *de novo* D1 protein synthesis (Nishiyama *et al.*, 2006).

In the long-term evolution of plants, many protective mechanisms were formed to quickly and effectively repair photodamaged PSII (Aro *et al.*, 2005; Takahashi and Badger, 2011). Several auxiliary factors, such as kinases, phosphatases, proteases, and DnaJ proteins, are involved in this repair cycle (Meurer *et al.*, 1998; Ma *et al.*, 2007; Chen *et al.*, 2010). DnaJ proteins are key components contributing to cellular protein homeostasis under stress conditions (Wang *et al.*, 2004). They function as molecular chaperones, alone or in association with heat-shock protein 70 (Hsp70) partners, and are involved in various essential cellular processes, including protein folding, degradation, and refolding (Hennessy *et al.*, 2005; Craig *et al.*, 2006). Since their discovery in *Escherichia coli* as 41kDa heat-shock proteins, DnaJ proteins have been found ubiquitously in all kingdoms of life (Georgopoulos *et al.*, 1980; Bukau and Horwich, 1998; Craig *et al.*, 2006). In general, DnaJ proteins contain one to four canonical domains (Silver and Way, 1993). Only the J-domain is completely conserved in all prokaryotic and eukaryotic groups. Through this domain, DnaJ proteins can interact with the ATPase domain of Hsp70 and hydrolyse ATP to ADP, facilitating client capture (Kampinga and Craig, 2010). Based on domain composition, the DnaJ family can be classified into three subtypes, with type III containing only a J-domain (Cheetham and Caplan, 1998). These subtypes are present in all major eukaryotic cell compartments, including the cytosol (Thomas and Baneyx, 1996), mitochondria (Voos and Rottgers, 2002), endoplasmic reticulum (Nicoll *et al.*, 2006), and chloroplasts (Orme *et al.*, 2001).

Chloroplasts are the structures in which photosynthesis mainly occurs. Previous studies have revealed that chloroplast-targeted DnaJ proteins participate in many processes that take place in the chloroplast, such as chloroplast development (Vitha *et al.*, 2003; Liu *et al.*, 2005, 2007; Albrecht *et al.*, 2008), phototropin-mediated chloroplast movement (Suetsugu *et al.*, 2005), and protein import and translocation (Chiu *et al.*, 2010). Three *Arabidopsis* chloroplast DnaJ proteins, AtJ8, AtJ11 and AtJ20, were found to be involved in optimization of photosynthetic reactions and stabilization of PSII complexes under high light stress (Chen *et al.* 2010). DnaJ proteins function alone or in association with Hsp70 partners. Hsp70B may protect PSII against damages through two distinct mechanisms: one that requires *de novo* D1 protein synthesis and one that does not (Schroda *et al.*, 2001). Whether the small DnaJ proteins function together with their Hsp70 partner, and whether the protective effect of these proteins on PSII requires *de novo* D1 protein synthesis, are interesting issues that need to be addressed.

Accordingly, we isolated a *c*hloroplast-targeted *D*na*J* protein (LeCDJ1) from tomato and submitted the sequence to GenBank under accession number GQ925907. This protein belongs to the simplest group of DnaJ proteins (type III) characterized specifically by the J-domain. The expression of *LeCDJ1* was induced by chilling stress. Overexpression of *LeCDJ1* in tomato alleviated chilling stress-induced photoinhibition, whereas its suppression increased chilling sensitivity.

## Materials and methods

### Plant materials, growth, and treatments

Three sense T_1_ transgenic tomato lines (S3, S7, and S14), wild tomato cultivar (*Solanum lycopersium* cv. Zhongshu 6), and three antisense T_1_ transgenic lines (A5, A11, and A13) were used as plant materials.

Seeds were sterilized, sown on Murashige and Skoog medium and incubated at 25 °C (light 16h/dark 8h) for 10 d. Some of the young seedlings were then exposed to 4 °C for 10 d and the growth performance was observed. The rest of the young seedlings were planted in plastic pots filled with sterilized soil and grown at 25/20 °C (day/night) with a 16h photoperiod, a photon ﬂux density (PFD) range of 500–600 μmol m^−2^ s^−1^, and a relative humidity range of 50–60% in a greenhouse. The plants were irrigated with Hoagland nutrient solution once a week. When the sixth leaf was fully expanded (approximately 2 months old), the plants were adapted in an illuminated incubation chamber (GXZ-260C) for 2 d before treatment. For chilling treatment in light, the plants were exposed to 4 °C in the illuminated incubation chamber with a PFD of approximately 200 μmol m^−2^ s^−1^ for 0, 3, 6, 12, and 24h, and then recovered at 25 °C with the same PFD for 2 and 4h. For chilling stress in darkness, the plants were grown at 25 °C in the same illuminated incubation chamber with all lights turned off for 18h and then exposed to 4 °C, still in darkness. For heat treatment, whole plants in pots were put in the illuminated incubation chamber at 42 °C for 0, 3, 6, 12, and 24h. For high light treatment, 2000 μmol m^−2^ s^−1^ light from daylight-type microwave sulfur lamps (MSL-1000; Youhe, Ningbo, China) was used. Salinity stress was performed by immersing the whole plants in 200mM NaCl solution for 0, 1, 2, and 3 d. Osmosic stress was administered by irrigating the seedlings with 20% polyethylene glycol (PEG) 6000. For oxidative treatment, the plant leaves were sprayed with 20mM H_2_O_2_. The treated plant leaves were harvested at an appropriate time, frozen in liquid nitrogen, and stored at –80 °C.

In another experiment, leaf discs were taken from plants exposed to the control growth condition. Half were immediately soaked in 3mM streptomycin (SM) solution in the dark, whereas the others were soaked in water to serve as the control. After approximately 3h, all discs were placed on the water surface at 4 °C. The water temperature was controlled by an RTE-211 water circulator (Thermo Fisher Scientific, Worcester, MA, USA).

### Isolation and sequencing of *LeCDJ1*

Total RNA was isolated using a total RNA isolation system (Tiangen, Beijing, China). For reverse transcription, 2 μg of total RNA was denatured at 70 °C for 5min. Next, 1 μl of avian myeloblastosis virus reverse transcriptase (Fermentas) was added, mixed brieﬂy, and incubated at 42 °C for 1h. The reaction was terminated at 70 °C for 10min.

For cloning of *LeCDJ1* from tomato, a pair of primers (JF and JR; Supplementary Table S1 at *JXB* online) was designed based on the *LeCDJ1* sequence (GenBank accession no. AK323422.1). PCR amplification was performed as follows: initial denaturation at 94 °C for 5min; 35 cycles of 94 °C for 50 s, 53 °C for 50 s, and 72 °C for 1min; ﬁnal extension at 72 °C for 10min; and reaction termination at 4 °C. The PCR ampliﬁcation products were cloned into the pMD-18T vector and then sequenced. All primers were synthesized by BioSune Biotechnology Ltd Co. (Jinan, China).

### RNA gel blot analysis

The total RNA (20 μg) of tomato was separated in a 1.2% agarose formaldehyde gel, transferred to a nylon membrane, and ﬁxed on the membrane by cross-linking with UV light. Pre-hybridization was performed at 65 °C for 12h. The 3′ partial cDNA of *LeCDJ1* labelled with [α-^32^P]dATP by random primed labelling (Prime-a-Gene-Labeling System; Promega) was used as the gene-speciﬁc probe. After 24h of hybridization, ﬁlters were washed subsequently in 2× SSC (1× SSC: 0.15M NaCl, 0.015M sodium citrate, pH 7) with 0.2% SDS and 0.2× SSC with 0.2% SDS at 42 °C. Autoradiography was performed at 80 °C.

### Quantitative real-time PCR (qRT-PCR) analysis

Total RNA extraction and reverse transcription were performed as previously above. qRT-PCR was performed on a Bio-Rad CFX96TM Real-time PCR System using SYBR Real Master Mix (Tiangen) with the following PCR thermal cycle conditions: denaturation at 95 °C for 30 s; and 40 cycles of 95 °C for 5 s, 58 °C for 10 s, and 68 °C for 10 s. *EF-1α* (GenBank accession no. X144491) was used as the actin. Template-free, negative, and single primer controls were established before the examination. The results are represented by three biological replicates (each with three technical replicates) for each sample, and a standard curve method was used for statistical analysis.

### Antibody preparation, total protein extraction, and western blot analysis

The coding region of *LeCDJ1* was subcloned into the pET-30a (+) vector between the *Bam*HI and *Sac*I sites. Expression and purification of the recombinant LeCDJ1 protein were carried out using a Ni-NTA agarose system according to the manufacturer’s instructions (Qiagen, Hilden, Germany). The purified recombinant protein was used to immunize the white mice and the obtained antiserum was then purified according to the Amersham Biosciences (Piscataway, NJ, USA) antibody purification protocol. The secondary antibody was a horseradish peroxidase-conjugated goat anti-mouse IgG (Santa Cruz Biotechnology, Santa Cruz, CA, USA). The primary antibody was used at a dilution of 1:500, whereas the secondary antibody was used at 1:5000.

Proteins were extracted from the leaves with extraction buffer (100mM HEPES, pH 7.5, 5mM EDTA, 5mM EGTA, 10mM dithiothreitol, 10mM Na_3_VO_4_, 10mM NaF, 1mM phenylmethanesulfonyl fluoride, 5mg ml^–1^ of leupeptin, 5mg ml^–1^ of aprotinin, 5% glycerol, 50mM β-glycerophosphate). After centrifugation at 15 000*g* for 30min at 4 °C, the supernatants were transferred into clean tubes, immediately frozen with liquid nitrogen, and then stored at –80 °C.

For western blotting, 20 μg of total plant proteins separated by SDS-PAGE were electrophoretically transferred to polyvinylidene fluoride (PVDF) membranes (Millipore, Billerica, MA, USA) and detected with antibody preparations. The protein content was determined by a dye-binding assay. Quantitative image analysis of protein levels was performed with a Tanon Digital Gel Imaging Analysis System (Tanon-4100; Shanghai Tanon Science and Technology Co., Shanghai, China).

### Subcellular localization of LeCDJ1

The N terminus of *LeCDJ1* was cloned and two DNA constructs (p35S-GFP and p35S-LeCDJ1-GFP) were prepared. Isolated *Arabidopsis* mesophyll protoplasts were transfected with the above two constructs as described by Shah *et al.* (2002) and examined by dual-channel confocal microscopy (LSM510 META; Zeiss, Germany). The GFP ﬂuorescence, red chloroplast autoﬂuorescence, and the bright-ﬁeld image of the protoplast were recorded simultaneously and compared. The potential co-localization of GFP ﬂuorescence and red chloroplast autoﬂuorescence was analysed further by checking for the presence of yellow signals in the superimposed images.

### Plant transformation and transgenic tomato plants identification

Full-length *LeCDJ1* cDNA was subcloned into the expression vector pBI121 downstream of the 35S cauliflower mosaic virus promoter. The constructs were then introduced into *Agrobacterium tumefaciens* LBA4404 by the freezing transformation method and verified by PCR and sequencing. Kanamycin-resistant transgenic tomato plants were generated using an *A. tumefaciens*-mediated leaf disk method. The DNA of the sense and antisense transgenic plants was extracted and used to amplify the target gene by PCR.

### Measurement of chlorophyll content

Ten-day-old young seedlings were incubated for 10 d at 4 °C. The whole plants were homogenized in 5ml of 80% acetone for 3 d and the homogenate was centrifuged at 3500*g* for 5min. The supernatant was retained and the absorbance was recorded at 663 and 646nm by UV spectrophotometry.

### Measurement of net photosynthetic rate (*P*_n_)

*P*_n_ was measured with a portable photosynthetic system (CIRAS-2; PP Systems, Herts, UK) under ambient CO_2_ conditions (360 μl l^−1^), a PFD of 800 μmol m^−2^ s^−1^, and a relative humidity of 80%. The illumination source was produced by light-emitting diodes. Before *P*_n_ measurement, the plants were kept for approximately 30min at 25 °C and at a PFD of 100 μmol m^−2^ s^−1^ to induce stomatal opening and then illuminated for approximately 15min at a PFD of 800 μmol m^−2^ s^−1^.

### Histochemical staining

Superoxide radical (O_2_^• −^) was detected visually by treating leaves with nitro blue tetrazolium (NBT) as described by Rao and Davis (1999). H_2_O_2_ was stained with 3′3′-diaminobenzidine (DAB) according to the method of Giacomelli *et al.* (2007). Trypan blue staining was performed as described by Choi *et al.* (2007).

### Measurement of O_2_^•−^ and H_2_O_2_ concentration

To measure the concentration of O_2_^•−^, leaves (0.5g) were ground with liquid nitrogen in a mortar and then transferred to a centrifugal tube. Next, 3ml of cold phosphate buffer (50mM, pH 7.8) was added, and the homogenate was centrifuged at 5000*g* for 10min at 4 °C. The supernatant with phosphate buffer (pH 7.8) and 10mM hydroxylammonium chloride was incubated at 25 °C for 20min, followed by the addition of 17mM *p*-aminobenzene sulfonic acid and 7mM α-naphthylamine. The mixture was then incubated at 25 °C for 20min and then centrifuged at 1500*g* for 5min. Finally, ethyl ether was added to the mixture. The water phase was used to determine the absorbance at 530nm.

For measurement of H_2_O_2_ concentration, leaves (0.5g) were ground with liquid nitrogen in a mortar and then transferred to a centrifugal tube. Subsequently, 3ml cold phosphate buffer (50mM, pH 6.8) was added. After centrifugation at 6000*g* for 15min, 3ml of supernatant and 1ml of 0.1% titanium sulfate in 20% (v/v) H_2_SO_4_ were added into a new tube, mixed, and then centrifuged again. The absorbance was determined at 410nm.

### Measurement of malondialdehyde (MDA) and the relative electric conductivity (REC)

For measurement of MDA content, leaves (0.5g) were ground in a cold mortar containing 10ml of 10% trichloroacetic acid. After centrifugation at 10 000*g* for 10min at 4 °C, 2ml of supernatant with 2ml of 0.6% thiobarbituric acid reagent [0.6% (m/v) thiobarbituric acid dissolved in 10% (m/v) trichloroacetic acid] was mixed, heated at 100 °C for 15min, and then quickly cooled and centrifuged at 5000*g* for 10min. The control contained 2ml of distilled water instead of MDA extract. Absorbance was determined at 450, 532, and 600nm. The MDA content was computed using a standard curve relating the MDA concentrations to absorbance.

Ten leaf discs (0.8cm diameter) from each line were placed in 20ml of distilled water, vacuumed for 30min, and then surged for 3h to measure the initial electric conductivity (*S*_1_). The materials were boiled for 30min and then cooled to room temperature to measure the final electric conductivity (*S*_2_). Distilled water was used as a blank control and its electric conductivity (*S*_0_) was measured. REC was calculated as REC=(S_1_ – S_0_)/(S_2_ – S_0_)×100.

### Measurement of the chlorophyll *a* fluorescence transient

The chlorophyll *a* fluorescence transient was measured using a Handy Plant Efficiency Analyzer (Hansatech Instruments, Norfolk, UK) with dark-adapted leaves under ambient CO_2_ conditions. The transient was induced by a red light of approximately 3000 μmol m^–2^ s^–1^ provided by an array of four light-emitting diodes (peak 650nm). The measurement protocol was as described by Zhang *et al.* (2012). The maximal photochemistry efficiency of photosystem II (PSII), *F*_v_/*F*_m_, was calculated as follows: *F*_v_/*F*_m_=1 – (*F*_o_/*F*_m_), where *F*_v_ is variable fluorescence, *F*_m_ is maximum fluorescence and *F*_o_ is minimum fluorescence (when all PSII centres are in the open state).

### Measurement of chlorophyll fluorescence

A FMS-2 pulse-activated modulation fluorometer (Hansatech, Cambridge, UK) was used to measure chlorophyll fluorescence. For the assay of photoinhibitory quenching (qI) and energy-dependent quenching (qE), plants were dark adapted for 12h before pre-stress *F*_m_ was measured. Leaves were then illuminated for 40min under actinic light (200 μmol m^−2^ s^−1^). This length of illumination was always found to be sufficient to reach a steady-state fluorescence yield and a maximum photosynthetic rate. A pulse of saturating light (3000 μmol m^−2^ s^−1^ for 700ms) was applied to determine the maximal fluorescence under actinic light (*F*_m_′). After this, the actinic light was switched off, and 40min later, the dark relaxation of fluorescence (*F*_mr_) was measured by applying saturating light pulses. qI and qE were calculated as follows: qI=*F*_m_/*F*_mr_ – 1, and qE=*F*_m_/*F*_m_′ – *F*_m_/*F*_mr_. Pre-stress *F*_m_ was used for the qI and qE calculation.

### Thylakoid membrane preparation and SDS-PAGE

For thylakoid membrane preparation, tomato leaves were homogenized in an ice-cold isolation buffer (400mM sucrose, 50mM HEPES, pH 7.8, 10mM NaCl, 2mM EDTA, and 2mM MgCl_2_) and filtered through three layers of pledget. The filtrate was centrifuged at 5000*g* for 10min. The thylakoid pellets were washed with isolation buffer, recentrifuged, and finally suspended in an isolation buffer. The thylakoid membrane proteins were then denatured and separated using a 15% polyacrylamide gradient gel containing 6M urea.

### Blue native PAGE (BN-PAGE) and western blotting

BN-PAGE was performed as described by Sun *et al.* (2007). After electrophoresis, the protein complexes were denatured with 15% methanol for 15min. The denatured protein complexes were then electroblotted onto PVDF membranes, probed with D1 antibody, and then visualized by the enhanced chemiluminescence method. X-ray films were scanned using an AlphaImager 2200 documentation system (Alpha Innotech).

### Yeast two-hybrid assays

Yeast two-hybrid assays were performed according to the Matchmaker^TM^ Gold Yeast Two-Hybrid System User Manual. The cpHsp70 coding region was amplified with cDNA from tomato seedlings and ligated to *Bam*HI/*Xhol*I-digested pGADT7 (Clontech). The LeCDJ1 coding region was amplified from a plasmid and then ligated to *Eco*RI/*Sal*I-digested pGBKT7 (Clontech). The expression vector pGADT7-cpHsp70 was co-transformed into yeast strain Y187 (Clontech) with pGBKT7-LeCDJ1 using the lithium acetate transformation method. Cells were plated onto selective medium without Leu and Trp (DDO). Putative transformants were transferred to selective medium without Leu, Trp, His, and adenine and supplemented with X-α-Gal and aureobasidin (QDO/X/A). The interactions between the p53 and T proteins, as well as between the Lam and T proteins, were used as positive and negative controls, respectively. Autoactivation was analysed by a growth experiment when the detected gene was co-transformed with the pGADT7 or pGBKT7 empty vector.

### Statistical analysis

Data points represent the mean ±standard deviation (SD) of three replications. Statistical significance of differences in the measured parameters between the wild type (WT) and transgenic plants was tested using the software in Excel. Significant differences in comparison with the control are indicated by *P<0.05 and **P<0.01.

## Results

### Expression patterns of *LeCDJ1* in tomato

**Fig. 1.**
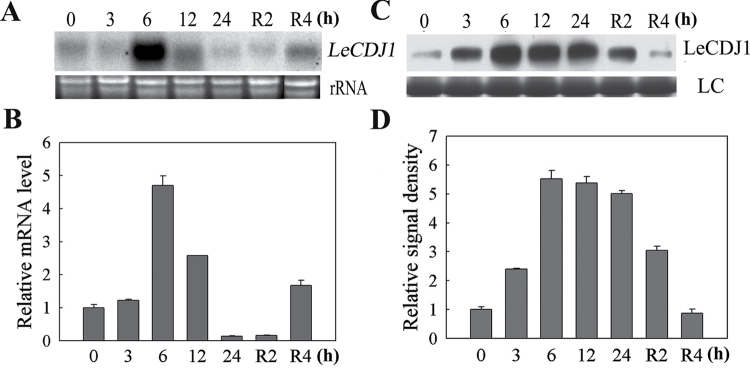
Responses of *LeCDJ1* to chilling stress in tomato. (A) RNA gel blot analysis of the expression of *LeCDJ1* in leaves treated at 4 °C in light (200 μmol m^−2^ s^−1^) for 0, 3, 6, 12, and 24h and then recovered at 25 °C for 2 and 4h. Ethidium bromide-stained rRNA is shown as a loading control. (B) qRT-PCR analysis of the response of *LeCDJ1* to chilling stress in light. The transcript level of *LeCDJ1* was normalized to *EF-1α* expression. Error bars represent the SD of triplicate reactions. (C) Western blot analysis of the LeCDJ1 protein levels in the leaves treated at 4 °C in light. LC, loading control (part of the Coomassie-stained total protein SDS-PAGE gel). (D) Quantitative image analysis of the protein content in (C) by Tanon Digital Gel Imaging Analysis System. The relative protein level of LeCDJ1 was normalized to the level at 0h.

*LeCDJ1* was isolated from a tomato cDNA library representing the expression patterns of genes affected by chilling. We first studied the expression of *LeCDJ1* in response to chilling using RNA gel blotting, qRT-PCR and western blotting. *LeCDJ1* was clearly induced by chilling treatment (Fig. 1A, B). The transcript reached a maximum level at 6h, decreased after 12h, and then recovered slightly to the original state after recovery at 25 °C for 4h. Moreover, chilling stress in darkness still induced *LeCDJ1* expression, although not as strongly as in light (Supplementary Fig. S1 at *JXB* online), suggesting that the response of *LeCDJ1* to chilling did not depend on the light. Western blot analysis showed that the protein signal became gradually stronger after treatment at 4 °C, but decreased slightly during the recovery period (Fig. 1C). The quantitative image analysis of protein content in Fig. 1C showed a similar profile (Fig. 1D).

**Fig. 2.**
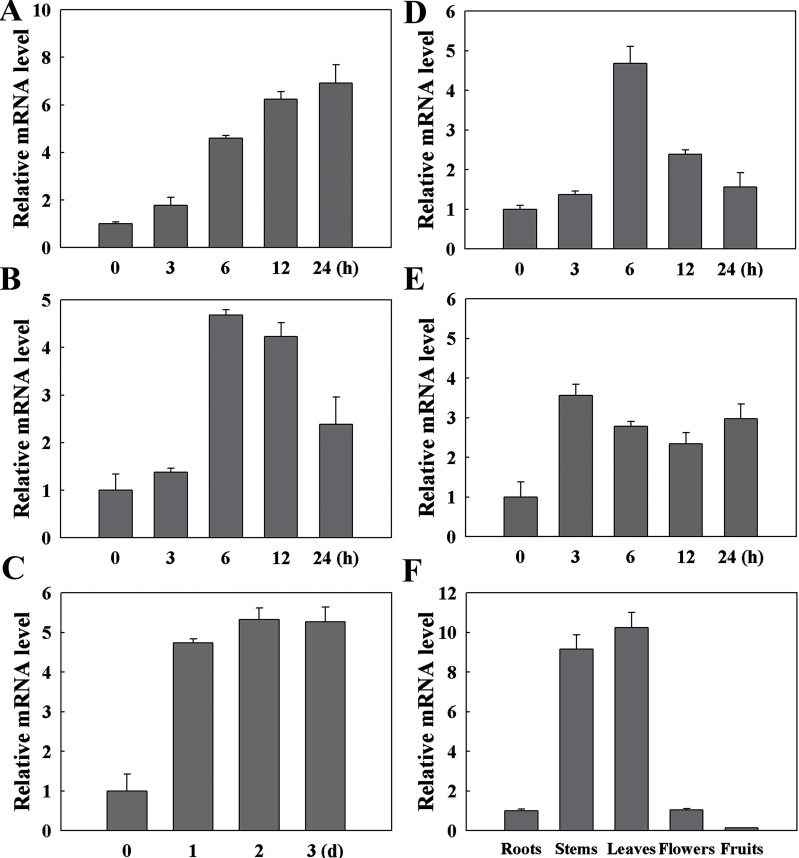
qRT-PCR analysis of the expression profiles of *LeCDJ1* in tomato. (A–E) Responses of *LeCDJ1* to 42 °C (A), high light (2000 μmol m^−2^ s^−1^ (B), 200mM NaCl (C), 20% PEG-6000 (D), and 20mM H_2_O_2_ (E). (F) Expression of *LeCDJ1* in the roots, stems, leaves, flowers, and fruits. The transcript level of *LeCDJ1* was normalized to *EF-1α* expression. Error bars represent the SD of triplicate reactions.

By qRT-PCR analysis, we detected the expression level of *LeCDJ1* under heat (42 °C), high light (2000 μmol m^−2^ s^−1^), salt (200mM NaCl), osmotic (20% PEG-6000) and oxidative (20mM H_2_O_2_) stresses at different time points (Fig. 2). All the tested stresses induced the expression of *LeCDJ1* to different extents. Heat stress induced the expression of *LeCDJ1* gradually (Fig. 2A). High light stress induced the expression during the first 12h, and the transcripts decreased at 24h (Fig. 2B). Treated with 200mM NaCl, the transcripts increased within 1 d, and remained high over a 3 d period (Fig. 2C). For 20% PEG treatment, transcripts reached a maximum level at 6h and then decreased (Fig. 2D). H_2_O_2_ treatments also led to a rapidly accumulation of the transcripts (Fig. 2E). These results suggested that *LeCDJ1* is involved in the response to various stresses.

We then examined the expression patterns of *LeCDJ1* in various organs. As shown in Fig. 2F, *LeCDJ1* was constitutively expressed in all organs examined and the transcript level in the leaves was approximately 10 times higher than that in the roots. Thus, the gene is expressed preferentially in chlorophyllous tissues.

### Chloroplast targeting of *LeCDJ1*

**Fig. 3.**
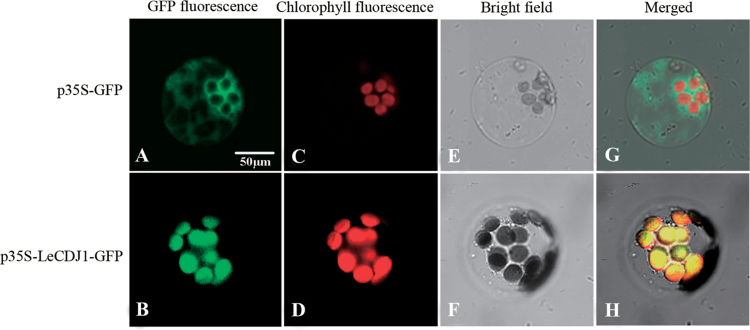
Intracellular targeting of LeCDJ1 in *Arabidopsis* protoplasts. (A, B) Green fluorescence of GFP (A) and the LeCDJ1–GFP fusion protein (B). (C, D) Red autofluorescence of chloroplasts. (E, F) Bright-field images of the protoplasts. (G, H) Merged images of (A), (C) and (E), and of (B), (D), and (F), respectively. Protoplasts were examined using dual-channel confocal microscopy (LSM510 META; Zeiss, Germany).

The programs ChloroP 1.1 (http://www.cbs.dtu.dk/services/ChloroP/) and TargetP 1.1 (http://www.cbs.dtu.dk/services/TargetP/) predicted that the first 45 aa of LeCDJ1 constituted the plastid target signals and that LeCDJ1 was probably a chloroplast protein (Supplementary Tables S2 and S3 at *JXB* online). To obtain direct experimental evidence, we performed targeting experiments *in vivo* in *Arabidopsis* protoplasts derived from leaf tissue. As shown in Fig. 3, green fluorescence in the individual protoplasts transfected with the control construct p35S-GFP (expressing the GFP coding sequence alone) was detected in the cytoplasm surrounding the chloroplasts and was not co-localized with the red autofluorescence of the chloroplasts. By contrast, when fused with LeCDJ1, the GFP signal was predominantly confined to the chloroplasts and co-localized with the red autofluorescence of the chloroplasts, suggesting that LeCDJ1 is a chloroplast protein.

In addition, amino acid sequence alignment between LeCDJ1 and DnaJ8 proteins from other plants showed that LeCDJ1 shared a high degree of sequence similarity to *Arabidopsis* AtDnaJ8 and pea PsJ8b (Supplementary Fig. S2 at *JXB* online). Both have been suggested to be a soluble stromal protein with a small portion peripherally associated with membranes where many key steps of PSII repair and reassembly processes take place (Chiu *et al.*, 2010).

### Identification of transgenic plants

**Fig. 4.**
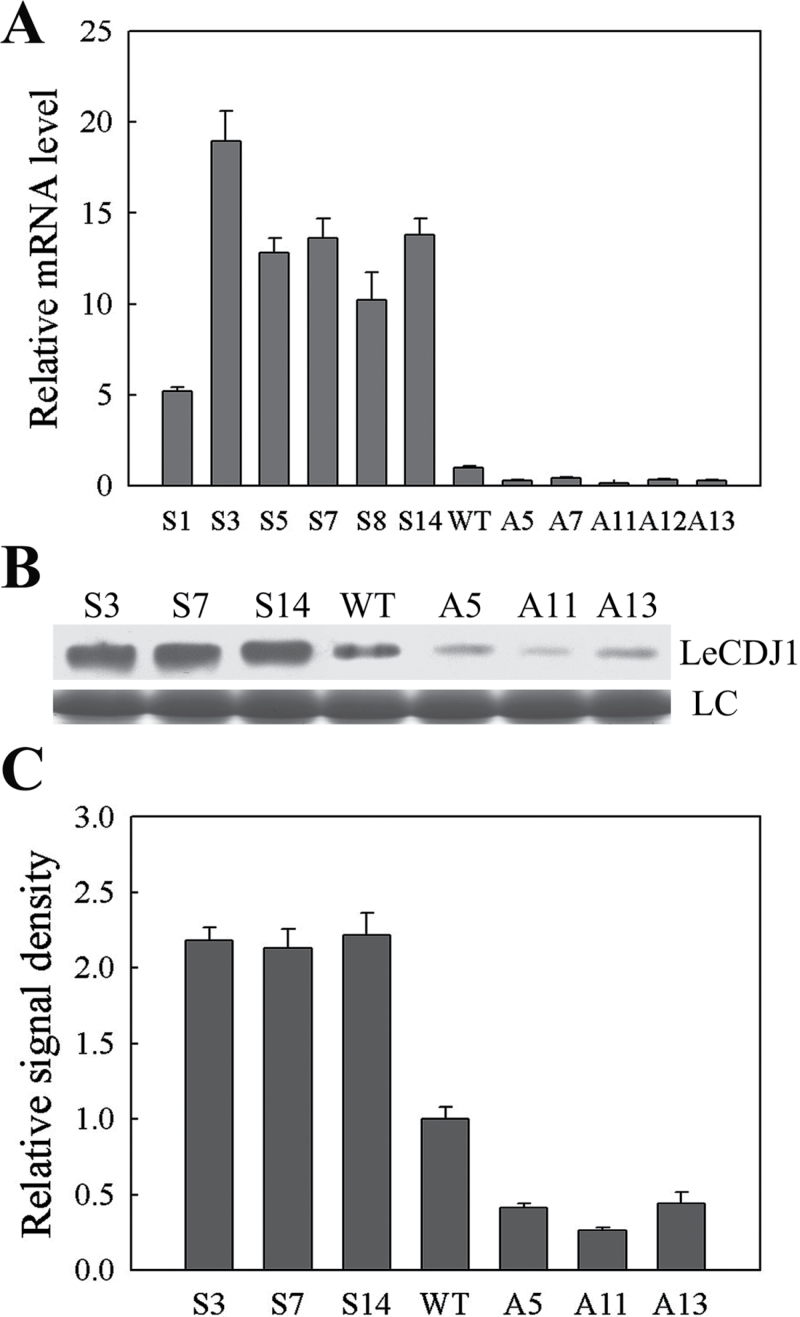
Identification of transgenic plants by qRT-PCR and western blotting. (A) Transcript levels of *LeCDJ1* in the WT and different transgenic lines. The transcript level of *LeCDJ1* was normalized to *EF-1α* expression. Error bars represent the SD of triplicate reactions. (B) LeCDJ1 protein levels in the WT and transgenic lines. LC, loading control (part of the Coomassie-stained total protein SDS-PAGE gel). (C) Quantitative image analysis of protein content in (B) using a Tanon Digital Gel Imaging Analysis System. The relative protein level of LeCDJ1 in the transgenic lines was normalized to that in the WT. Two-month-old plants were used.

A total of 31 (16 sense lines, 15 antisense lines) individual kanamycin-resistant tomato transgenic lines (T_0_) were harvested from tissue culture. The progeny obtained from T_0_ were named T_1_. Six sense (S1, S3, S5, S7, S8, and S14) and five antisense (A5, A7, A11, A12, and A13) T_1_ lines were selected for qRT-PCR. Compared with WT, the relative *LeCDJ1* mRNA level in the examined sense lines increased by 5.3-, 19.7-, 13.3-, 14.1-, 10.5-, and 14.3-fold, whereas that in the antisense lines decreased by 0.30-, 0.43-, 0.14-, 0.34- and 0.31-fold, respectively (Fig. 4A). Among these lines, S3, S7, S14, A5, A11, and A13 were selected for western blot analysis. The pattern of LeCDJ1 protein levels was similar to that of mRNA levels (Fig. 4B, C). Hence, S3, S7, S14, A5, A11, and A13 were selected for the following physiological measurements.

### *LeCDJ1* overexpression enhanced chilling stress tolerance

**Fig. 5.**
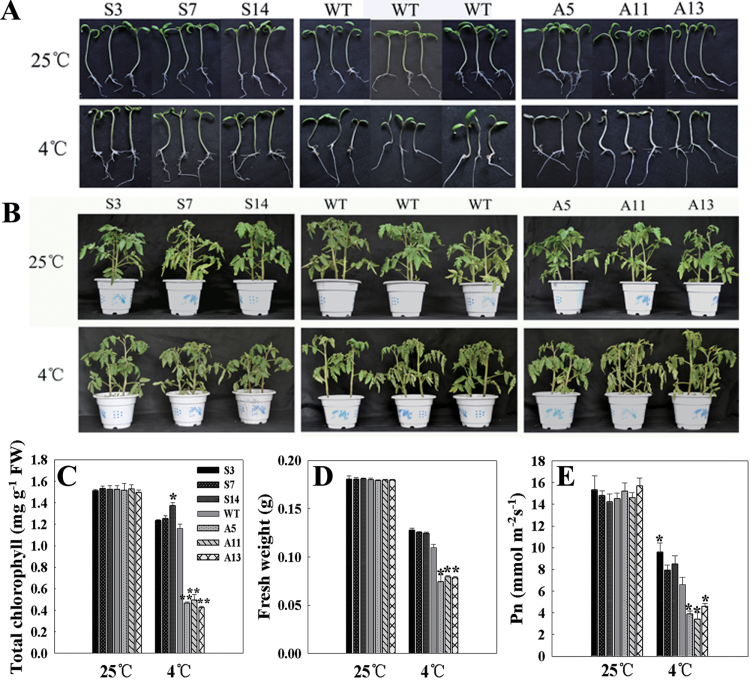
Growth analysis of young seedlings (10 d old) and grown plants (2 months old). (A) Growth performance of the young seedlings. (B) Growth performance of the grown plants. The top panel represents plants grown at 25 °C, and the bottom panel represents plants treated at 4 °C for 10 d (10-d-old young seedlings) or 24h (2-month-old grown plants). (C) Total chlorophyll content in the young seedlings. (D) Fresh weight of the young seedlings. (E) *P*_n_ in the grown plants. Plants were treated at 4 °C, and *P*_n_ was measured at 25 °C under ambient CO_2_ conditions (360 μl l^−1^). Before *P*_n_ measurement, tomato plants were kept for approximately 30min at 25 °C with a PFD of 100 μmol m^−2^ s^−1^ to induce stomatal opening and then illuminated for approximately 15min at a PFD of 800 μmol m^−2^ s^−1^. For (C) to (E), each column represents the mean ±SD of three replicates. Statistically significant differences in comparison with the control are indicated: *P<0.05 and **P<0.01, respectively.

The chilling stress tolerance of the plants was determined by observing the growth performance of the young seedlings (approximately 10 d old) and grown plants (approximately 2 months old). Under normal conditions, both grew well and showed no significant difference in phenotype and physiological traits (Fig. 5). After treatment at 4 °C (young seedlings for 10 d and grown plants for 24h), the growth of all plants was more or less suppressed. The grown plants showed little difference between sense, WT, and antisense plants (Fig. 5B). However, suppression of the growth of the young seedlings was less serious in the sense plants and more serious in the antisense plants, compared with WT. The leaves of the antisense young seedlings showed photobleaching, whereas most leaves of the sense lines remained green (Fig. 5A). Accordingly, the sense plants showed higher chlorophyll contents (approximately 83.6% that of the untreated WT plants, on average), whereas the antisense plants had a larger drop (approximately 30.3%, on average), compared with WT (approximately 76.3%) (Fig. 5C). Similarly, the fresh weight of WT plants was 0.111±0.0041g, which was lighter than S3 (0.1274±0.00261g), S7 (0.1254±0.00106g), and S14 (0.1245±0.00114g) plants, but heavier than A5 (0.0741±0.0041g), A11 (0.0793±0.00105g), and A13 (0.0782±0.00149g) plants (Fig. 5D).

After chilling treatment, the *P*_n_ of both WT and transgenic grown plants markedly decreased. However, the decrease in the antisense plants was also more serious than that in the sense plants (Fig. 5E). These results indicated that the transgenic plants with higher *LeCDJ1* expression levels had higher chilling tolerance, whereas those with lower *LeCDJ1* expression levels had higher chilling sensitivity.

### *LeCDJ1* overexpression alleviates reactive oxygen species (ROS) accumulation under chilling stress

**Fig. 6.**
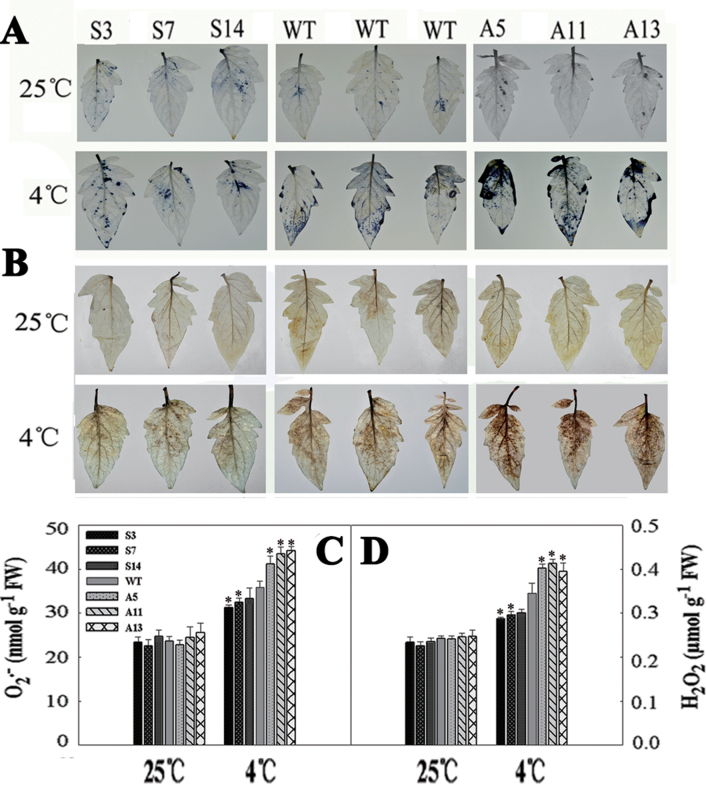
O_2_^• −^ and H_2_O_2_ analysis in the WT and transgenic plants. (A) NBT staining for O_2_^• −^. (B) DAB staining for H_2_O_2_. The top panel represents plants grown at 25 °C and the bottom panel represents plants treated at 4 °C for 12h. (C) O_2_^• −^ content in tomato plants. (D) H_2_O_2_ content in tomato plants. The experiment was repeated three times with similar results. The data are represented as means ±SD of three biological replicates (*P<0.05). Two-month-old grown plants were used. (This figure is available in colour at *JXB* online.)

Chilling stress accelerates ROS generation, so we analysed intracellular levels of O_2_^• −^ and H_2_O_2_ by NBT and DAB staining, respectively. Prior to treatment, both O_2_^• −^ and H_2_O_2_ accumulation was low, with no significant difference between the WT and transgenic plants. After chilling treatment for 12h, NBT staining for O_2_^• −^ showed that the blue polymerization product due to O_2_^• −^ accumulation increased, especially in WT and antisense plants (Fig. 6A). The colour was darkest in the antisense plants. Similarly, DAB staining showed that H_2_O_2_ accumulation also increased after chilling stress, and the accumulation was less in S3, S7, and S14 plants, but more in A5, A11, and A13 plants, than in WT (Fig. 6B). Quantitative analysis of O_2_^• −^ and H_2_O_2_ showed a similar result (Fig. 6C, D). These results suggested that *LeCDJ1* overexpression alleviates the accumulation of O_2_^• −^ and H_2_O_2_.

### *LeCDJ1* overexpression alleviates membrane damage under chilling stress

**Fig. 7.**
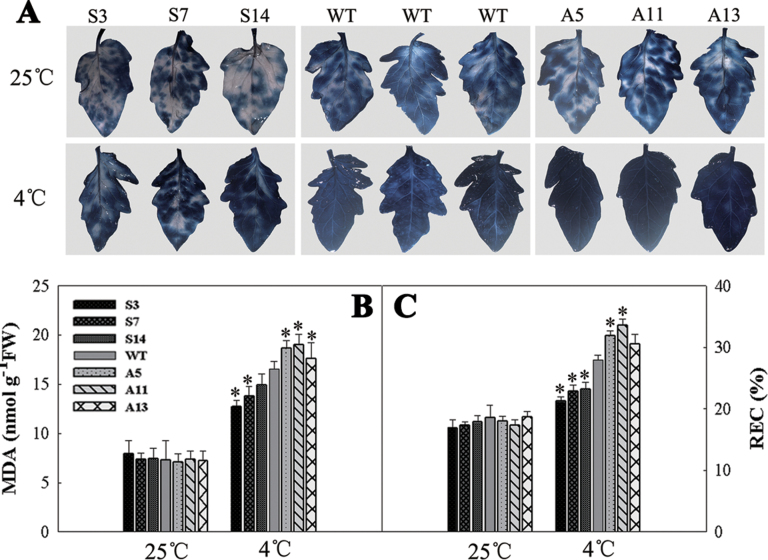
Membrane damage analysis in the WT and transgenic plants. (A) Trypan blue staining. The top panel represents plants grown at 25 °C and the bottom panel represents plants treated at 4 °C for 12h. (B) MDA content in the WT and transgenic plants. (C) REC in the WT and transgenic plants. Three independent experiments are shown as means ±SD. Asterisks indicate significant differences from WT of the same treatment (*P<0.05). Two-month-old grown plants were used. (This figure is available in colour at *JXB* online.)

As the primary target of chilling stress, membranes are particularly susceptible to ROS-initiated lipid peroxidation reactions. Trypan blue staining showed that all plants had a similar level of blue marks under normal growth condition. However, after 12h of chilling treatment, the antisense lines exhibited darker blue marks than the WT and sense plants (Fig. 7A). For confirmation, MDA accumulation and REC, which have been reported as indicators for the membrane damage, were determined. As shown in Fig. 7B and C, both MDA accumulation and REC increased after chilling stress. In addition, the increases were more obvious in the antisense lines and less obvious in the sense lines. These results suggested that, compared with WT, membrane damage was less serious in the sense plants and more serious in the antisense plants.

### *LeCDJ1* overexpression alleviates photoinhibition of PSII under chilling stress

**Fig. 8.**
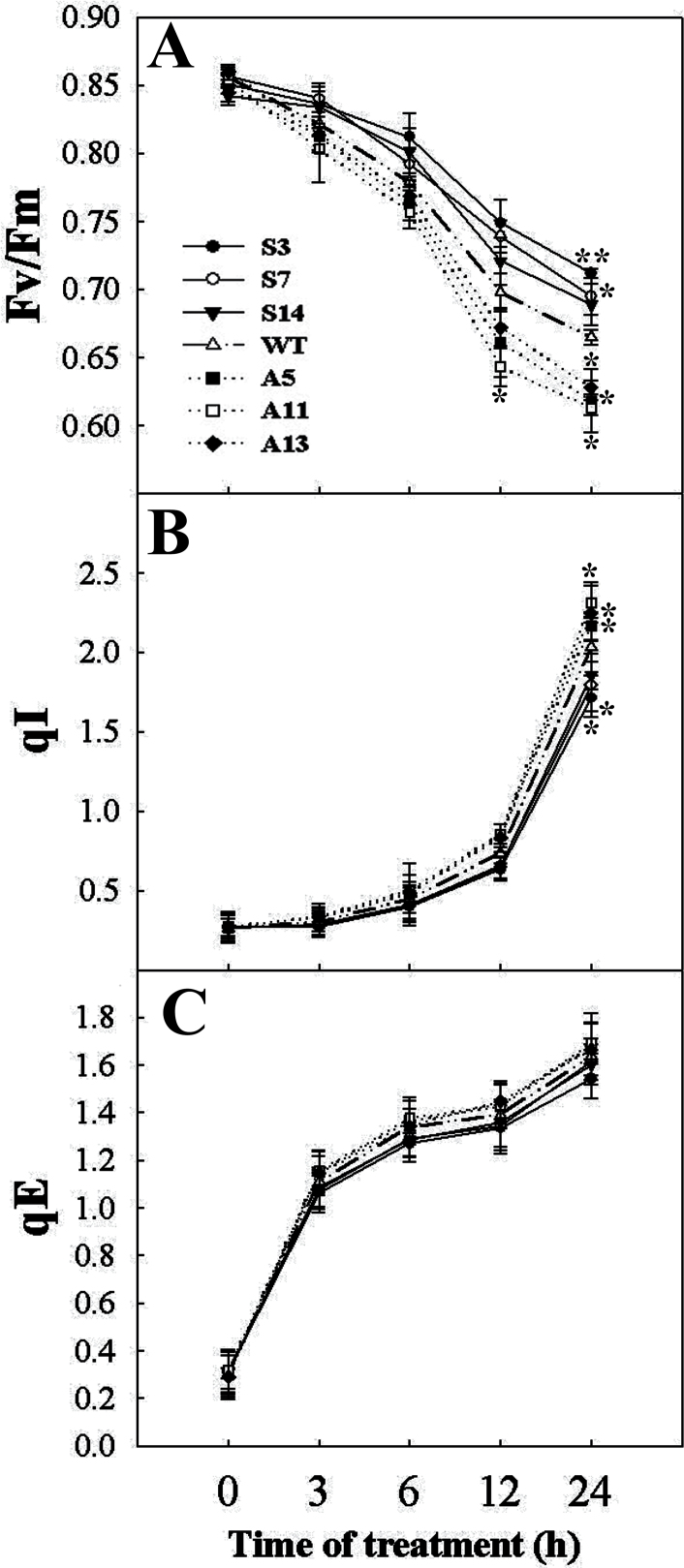
Changes in *F*_v_/*F*_m_ (A), qI (B), and qE (C) under chilling treatment in tomato plants. Plants grown at 25 °C in a greenhouse were exposed to 4 °C in the chambers for 0, 3, 6, 12, and 24h in the light (200 μmol m^−2^ s^−1^). Plants were dark adapted for 30min before measurement. The values are represented as means ±SD of three independent experiments (*P<0.05). Two-month-old grown plants were used.

*F*_v_/*F*_m_ was used to measure PSII photoinhibition. Only a slight difference was noted between the WT and transgenic lines before treatment. After chilling stress, *F*_v_/*F*_m_ decreased slightly in the sense plants, but decreased markedly in the antisense plants, compared with WT, suggesting that *LeCDJ1* overexpression decreases chilling stress-induced PSII photoinhibition (Fig. 8A).

The decrease in *F*_v_/*F*_m_ during chilling stress has various explanations: for instance, a change in qI and qE. During and after chilling treatment, qI, part of which is due to D1 damage, was lower in the sense plants and higher in the antisense plants, compared with WT (Fig. 8B). The substantial increases in qI indicated that the antisense plants experienced more photoinhibition than the WT and sense plants under chilling stress (Dodd *et al.*, 1998). Interestingly, qE values among the different tested lines were not significantly different, indicating that the lower *F*_v_/*F*_m_ in the antisense plants was most possibly caused by the higher qI (Fig. 8C).

### *LeCDJ1* overexpression alleviates photoinhibition of PSII whether in the presence of organelle protein synthesis or not

**Fig. 9.**
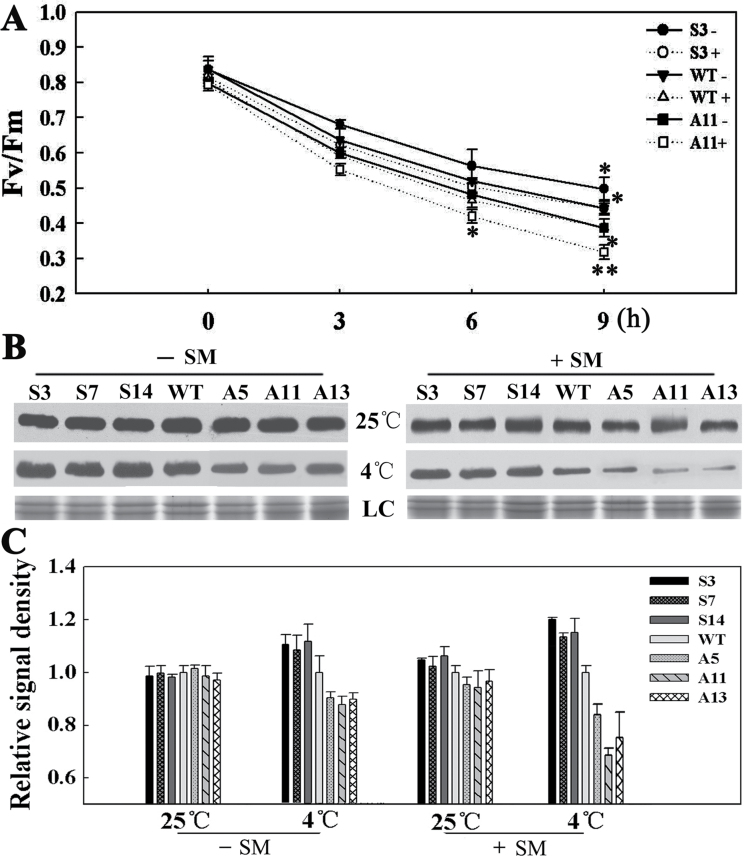
(A, B) Effect of SM and chilling stress on *F*_v_/*F*_m_ (A) and D1 protein content (B). ‘–’ indicates untreated plants, and ‘+’ indicates plants treated with SM. Leaf discs were taken from plants grown at 25 °C, soaked in 3mM SM solution in the dark for 3h and exposed to 4 °C for 0, 3, 6, and 9h. Leaf discs were dark adapted for 30min before measurement. The data are represented as means ±SD of three biological replicates (*P<0.05; **P<0.01). LC, loading control (part of the Coomassie-stained thylakoid membrane protein SDS-PAGE gel). (C) Quantitative image analysis of the D1 protein content in (B) using a Tanon Digital Gel Imaging Analysis System. The relative protein level of D1 in the transgenic lines was normalized to that in the WT. Two-month-old grown plants were used. The data are shown as means ±SD.

SM, an inhibitor of organellar translation that can inhibit *de novo* D1 protein synthesis, was used to investigate whether the protective effect of LeCDJ1 on PSII was dependent on D1 protein synthesis. *F*_v_/*F*_m_ in leaf discs treated with water or SM at 25 °C decreased slightly and showed no obvious difference between the tested lines (data not shown). After chilling stress, *F*_v_/*F*_m_ in the plants decreased markedly. It was also higher in S3 and lower in A11 leaves, compared with WT, especially in the presence of SM (Fig. 9A). For example, after chilling treatment for 9h, *F*_v_/*F*_m_ in S3, WT, and A11 leaves decreased to approximately 60, 53, and 48% of the original value, respectively. In the presence of SM, an approximately 48.1% decrease in *F*_v_/*F*_m_ was observed in S3 leaves, whereas 59.9% was found in A11 leaves. Interestingly, *F*_v_/*F*_m_ in the SM-treated S3 leaves was higher than that in the SM-untreated A11 leaves during chilling treatment. Given that this higher portion was not due to *de novo* D1 protein synthesis, the protective effect of LeCDJ1 on PSII, at least partially, was not dependent on D1 protein synthesis.

To investigate whether the impaired PSII function was associated with the changes in D1 protein levels, we performed western blot analysis of thylakoid membrane preparations with equal amounts of chlorophyll. As shown in Fig. 9B, D1 protein levels showed no obvious difference between the tested lines under normal condition. However, after 12h of chilling treatment, D1 protein showed a stronger signal in the sense plants, and a weaker signal in the antisense plants, compared with WT. As a chloroplast translation inhibitor, SM further decreased the D1 protein content in all strains. However, this decrease was more obvious in the antisense plants than in the sense plants. The quantitative image analysis of protein content in Fig. 9B further proved our previous findings that the D1 protein content was more in the sense plants and less in the antisense plants, whether in the presence of SM or not (Fig. 9C). These results indicated that LeCDJ1 has a protective effect on D1 protein and this effect is at least partially not dependent on D1 protein *de novo* synthesis.

### *LeCDJ1* overexpression stabilizes the thylakoid protein complexes

**Fig. 10.**
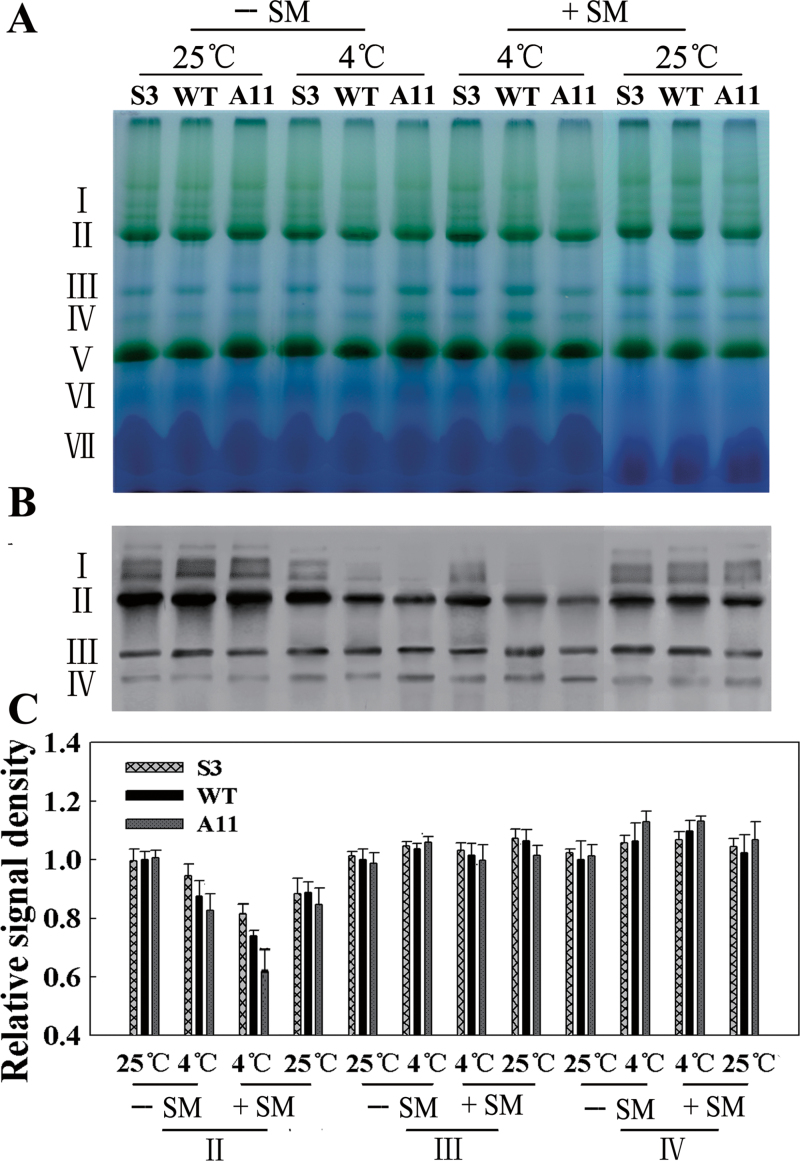
Stability of PSII complexes analysed by BN-PAGE and western blotting. (A) BN-PAGE gel. Thylakoid membranes (10 μg of chlorophyll) were solubilized with 1% *n*-dodecyl β-d-maltoside and separated by BN gel electrophoresis. The positions of protein complexes representing PSII–LHCII supercomplexes (band I), monomeric PSI and dimeric PSII (band II), monomeric PSII (band III), CP43-free PSII (band IV), trimeric LHCII/PSII reaction centre (band V), monomeric LHCII (band VI), and unassembled proteins (band VII). SM was added (+) or not (–) to a final concentration of 3mM. (B) Immunodetection of thylakoid protein complexes separated by BN-PAGE with anti-D1 antibody. The protein complexes were denatured with 15% methanol for 15min before being electroblotted onto PVDF membranes. (C) Quantitative image analysis of the protein content in (B) using a Tanon Digital Gel Imaging Analysis System. The relative protein level of D1 was normalized to that in the WT grown at 25 °C in the absence of SM. Two-month-old grown plants were used.

Altered *LeCDJ1* expression affected PSII activity (Fig. 8) and D1 protein levels (Fig. 9). Hence, we evaluated putative alterations in thylakoid protein complexes and performed BN-PAGE analysis. Seven major bands labelled I to VII were resolved, apparently representing PSII–LHCII supercomplexes (band I), monomeric PSI and dimeric PSII (band II), monomeric PSII (band III), CP43-free PSII (band IV), trimeric LHCII/PSII reaction centre (band V), monomeric LHCII (band VI), and unassembled proteins (band VII). Few differences were apparent in the BN gel, except that the amount of PSII–LHCII supercomplexes (band I) was a little more in S3 than in A11 plants, after chilling stress in the presence of SM (Fig. 10A). Immunoblotting of the BN gel with D1 antibody showed more clearly the decrease in PSII–LHCII supercomplexes (band I) in the tested lines after chilling stress (Fig. 10B). S3 plants showed a smaller decrease, while A11 plants showed a larger decrease, especially in the presence of SM. The PSII–LHCII supercomplexes completely disappeared from A11 plants in the presence of SM. Moreover, the amount of PSII dimers and monomers (band II) was also decreased, especially in WT and A11 plants, after chilling treatment. The monomeric PSII (band III) showed few changes among the tested lines after treatment. The CP43-free PSII (band IV) increased slightly after chilling stress, indicating that PSII repair was occurring. The quantitative image analysis of protein content in Fig. 10B showed the same trend (Fig. 10C). These findings indicated that LeCDJ1 provides a stabilizing force for these protein complexes.

In the present study, determination of an interaction between LeCDJ1 and these protein complexes was also attempted. However, western blotting of the BN gel with LeCDJ1 antibody showed no positive signal (data not shown). This finding may be attributed to the fact that their interaction was not strong enough to bear the solubilization of *n*-dodecyl β-d-maltoside.

### Interaction between LeCDJ1 and a chloroplast Hsp70

**Fig. 11.**
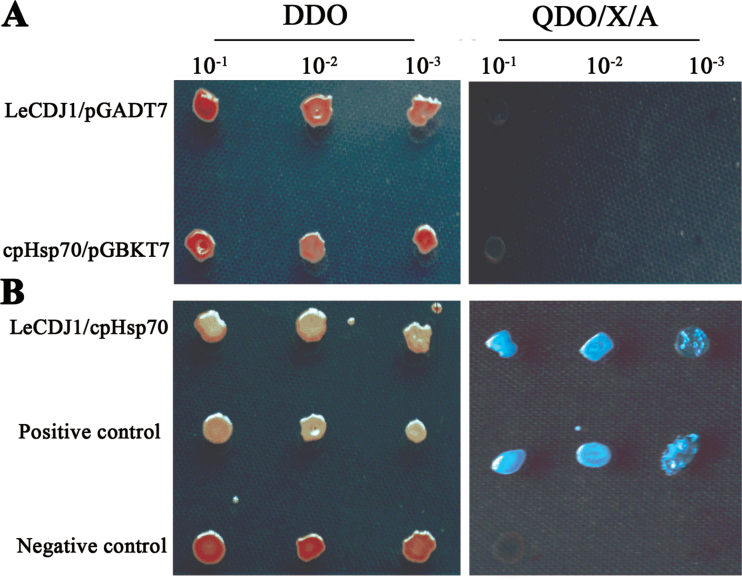
Interaction between LeCDJ1 and cpHsp70. (A) Test for autoactivation. (B) Yeast two-hybrid analysis of the interaction between LeCDJ1 and cpHsp70. An interaction between the p53 and T proteins was used as positive control (middle panel) and interaction between the Lam and T proteins (bottom panel) was used as negative control. Yeast strains harbouring the indicated plasmids were grown on selective medium without Leu and Trp (DDO; left panel) or selective medium without Leu, Trp, His, and adenine and with X-α-Gal and aureobasidin (QDO/X/A; right panel).

To date, DnaJ proteins are known to act as important chaperones for Hsp70. Hence, we postulated that LeCDJ1 interacts with a specific Hsp70(s). According to its chloroplast localization (Fig. 3), the partner of LeCDJ1 was anticipated to be a tomato chloroplast-located protein. We then searched the NCBI database (http://www.ncbi.nlm.nih.gov) for ‘*Solanum lycopersicum* chloroplast Hsp70’ and identified a cDNA (GenBank accession no EU195057.1) encoding a chloroplast Hsp70. The programs ChloroP 1.1 and TargetP 1.1 also predicted that the isolated Hsp70 was probably a chloroplast protein (Supplementary Tables S2 and S3). A yeast two-hybrid assay was then performed, and the capacity of the yeast to grow on QDO/X/A was used as an interaction marker. All yeast transformants grew normally on DDO. The yeast co-transformed with LeCDJ1 and pGADT7 or cpHsp70 and pGBKT7 empty vectors did not grow on QDO/X/A (Fig. 11A), indicating that none of the detected genes was autoactivated. However, when LeCDJ1 was co-transformed with cpHsp70, blue colonies growing on QDO/X/A were found, indicating an interaction between LeCDJ1 and cpHsp70 (Fig. 11B).

## Discussion

DnaJ proteins act as molecular chaperones re-establishing normal protein conformation and cellular protein homeostasis under stress conditions (Wang *et al.*, 2004). They are often referred to as cellular stress sensors and their expression is induced by heat, high light, methyl viologen and cold (Piippo *et al.*, 2006; Scarpeci *et al.*, 2008; Rajan and D’Silva, 2009). In this study, the expression of *LeCDJ1* was induced by chilling, whether in light or darkness, suggesting that the response of *LeCDJ1* to chilling did not depend on light (Fig. 1, Supplementary Fig. S1). We also established that *LeCDJ1* was involved in the response to heat, high light, NaCl, PEG and H_2_O_2_ (Fig. 2). This elevated expression may be attributed to the enhanced protection of plants against the damaging effect of these environmental stresses. In fact, the roles of DnaJ proteins in response to environmental stresses have already been established by several studies (Li *et al.*, 2007; Yang *et al.*, 2009; Chen *et al.*, 2010; Zhou *et al.*, 2012; Bekh-Ochir *et al.*, 2013). However, direct physiological evidence that supports the involvement of these proteins in chilling tolerance is still lacking. The data of the present study revealed the role of LeCDJ1, a type III chloroplast-targeted DnaJ protein (Fig. 3), in the maintenance of PSII activity under chilling stress.

Chilling stress inhibits PSII activity in several ways, for example lowering the rate of photosynthesis, blocking the PSII photosynthetic electron transport, and accelerating the generation of ROS, as well as damaging the membrane lipids and photosynthetic pigment complex systems (Suzuki *et al.*, 2008). Several lines of evidence present in this study indicated that LeCDJ1 was involved in the maintenance of PSII activity under chilling stress. After chilling treatment, the sense plants exhibited not only higher chlorophyll content, fresh weight, and *P*_n_, but also lower membrane damage (Figs 5 and 7). DnaJ proteins are involved in ROS level regulation in the chloroplast (Chen *et al.*, 2010) and mitochondria (Zhou *et al.*, 2012). In the present experiment, compared with WT, the S3, S7, and S14 plants maintained lower O_2_^• –^ and H_2_O_2_ levels, whereas the A5, A11, and A13 plants accumulated more O_2_^• –^ and H_2_O_2_, suggesting that LeCDJ1 could help reduce the ROS level (Fig. 6). Moreover, the photochemical efficiency of PSII, the *F*_v_/*F*_m_ ratio, in the sense lines decreased less markedly than that in the WT and antisense plants, suggesting that *LeCDJ1* overexpression decreased PSII photoinhibition (Fig. 8A). Accordingly, the lower increases in qI also indicated that the sense plants experienced less photoinhibition than WT and antisense plants under chilling stress (Fig. 8C). Thus, as discussed above, LeCDJ1 is involved in plant chilling tolerance and its overexpression alleviates PSII photoinhibition under chilling stress.

Additional lines of evidence supporting the roles of LeCDJ1 in PSII maintenance included the increased amounts of PSII reaction centre D1 protein and the increased stability of the PSII protein complexes in the sense plants (Figs 9B and 10). PSII is particularly prone to photo-oxidative damage as the water splitting reaction catalysed by this complex inevitably leads to the generation of ROS that damage the complex. Among the PSII proteins, the PSII reaction centre D1 protein is the main target of damage. Plants compromised in PSII photosynthetic activity have low levels of D1 protein (Suorsa *et al.*, 2004). After chilling treatment for 12h, the relative abundances of D1 protein were slightly higher in the sense plants and lower in the antisense plants, suggesting that PSII photosynthetic activity was compromised in the antisense plants (Fig. 9B, C). In addition, the PSII–LHCII supercomplexes and PSII dimers and monomers were also increased in the sense plants and reduced in the antisense plants at this time point, suggesting that LeCDJ1 may provide a stabilizing force for these protein complexes (Fig. 10B, C). Thus, our preliminary results suggest the importance of LeCDJ1 in the maintenance of PSII activity under chilling stress.

The process of PSII maintenance that might involve LeCDJ1 was also determined in this study. It is known that the extent of PSII photoinhibition is associated with a balance between the rate of PSII damage and repair (Takahashi and Murata, 2006). The main target of PSII damage is the PSII reaction centre D1 protein, whereas PSII repair requires D1 protein degradation, synthesis, and then reassembly into the PSII complex (Nishiyama *et al.*, 2006). To distinguish between the two processes, SM, an inhibitor of organellar translation (inhibitor of *de novo* D1 protein synthesis), was used. The sense plants displayed a higher *F*_v_/*F*_m_, D1 protein content, and PSII protein complex stability whether in the presence of SM or not (Figs 9 and 10). Interestingly, after chilling stress, *F*_v_/*F*_m_, D1 protein content, and PSII protein complex stability in the SM-treated sense lines were higher than those in the SM-untreated antisense plants (Figs 9 and 10). Given that this higher proportion was not caused by *de novo* D1 protein synthesis, the protective effect of LeCDJ1 on PSII was, at least partially, not dependent on D1 protein synthesis. The contribution of plant chaperone proteins, such as heat-shock proteins, to the stability of proteins has been shown by studies *in vivo* (Low *et al.*, 2000; Basha *et al.*, 2010) and *in vitro* (Lee and Vierling, 2000; Basha *et al.*, 2004). Considering the general function of DnaJ proteins as chaperone proteins (Walsh *et al.*, 2004), an effect of LeCDJ1 on PSII that does not depend on *de novo* D1 protein synthesis could be explained by the possible role of LeCDJ1 in D1 protein conformation protection.

DnaJ proteins act as co-chaperones of the Hsp70 machinery and are important in the stimulation of Hsp70s ATPase activity, thereby stabilizing its interaction with client proteins (Mayer and Bukau, 2005; Kampinga and Craig, 2010). Subsequently, members of the DnaJ proteins have been found to function as molecular chaperones, alone or in association with Hsp70 partners. A chloroplast Hsp70 (cpHsp70) in tomato, which could interact with LeCDJ1 directly, was identified through a yeast two-hybrid assay in this study (Fig. 11). Therefore, the LeCDJ1/cpHsp70 machinery may work together in PSII maintenance. In fact, the involvement of Hsp70B as a stabilizing force of the thylakoid membrane protein complexes is not without precedent (Schroda *et al.*, 1999; Yokthongwattana *et al.*, 2001). In general, DnaJ proteins drive the functional diversity of Hsp70s through two mechanisms. First, they interact with Hsp70 directly and stimulate the ATPase activity necessary for the stable binding of Hsp70 to their protein substrate (Bukau and Horwich, 1998). Secondly, DnaJ proteins localized to a particular site in a cellular compartment could be maintained at a high local concentration, thus targeting Hsp70 to particular client proteins at these sites (Nakatsukasa *et al.*, 2008). The present data are insufficient to depict the definite role of LeCDJ1 in the Hsp70/DnaJ protein machinery. The effect of LeCDJ1 on the ATPase-affecting conformational changes in cpHsp70 should be determined to differentiate ultimately the two possible ways.

In conclusion, overexpression of *LeCDJ1 in vivo* resulted in increased tolerance of PSII against chilling stress, whereas suppression of *LeCDJ1* increased PSII chilling sensitivity. Based on our data and previous knowledge, we postulate that the protective effect of LeCDJ1 on PSII is, at least partially, not dependent on *de novo* D1 protein synthesis and perhaps occurs by conformation protection of the D1 protein, together with cpHsp70.

## Supplementary data

Supplementary data are available at *JXB* online.

Supplementary Fig. S1. Response of *LeCDJ1* to chilling stress in darkness.

Supplementary Fig. S2. Amino acid sequence alignment between LeCDJ1 and DnaJ8.

Supplementary Table S1. Specific primers used in this study.

Supplementary Table S2. Prediction of subcellular localization of LeCDJ1 and cpHsp70 by the software program ChloroP 1.1.

Supplementary Table S3. Prediction of subcellular localization of LeCDJ1 and cpHsp70 using the software program TargetP 1.1.

Supplementary Data

## Abbreviations:

BN-PAGE: blue native polyacrylamide gel electrophoresis
DAB: 3′3′-diaminobenzidine
*F*_v_/*F*_m_: the maximal photochemistry efficiency of PSII
MDA: malondialdehyde
NBT: nitro blue tetrazolium
PEG: polyethylene glycol
PFD: photon ﬂux density
*P*_n_: net photosynthetic rate
PSII: photosystem II
PVDF: polyvinylidene fluoride
qE: energy-dependent quenching
qI: photoinhibitory quenching
qRT-PCR: quantitative real-time PCR
REC: relative electric conductivity
ROS: reactive oxygen species
SD: standard deviation
SM: streptomycin
WT: wild type.
