# Supplementary material for: A chloroplast-targeted DnaJ protein contributes to maintenance of photosystem II under chilling stress
Source: J Exp Bot. 2013 Nov 13;65(1):143–58. doi: 10.1093/jxb/ert357 (PMC3883286; doi:10.1093/jxb/ert357)
Supplement: Supplementary Data [file supp_65_1_143__index.html]

A chloroplast-targeted DnaJ protein contributes to maintenance of photosystem II under chilling stress — A chloroplast-targeted DnaJ protein contributes to maintenance of photosystem II under chilling stress — Supplementary Data 

# A chloroplast-targeted DnaJ protein contributes to maintenance of photosystem II under chilling stress

## Supplementary Data

Data files

**Files in this Data Supplement:**

- Supplementary Data - Supplementary Data
