# Supplementary material for: A chloroplast-targeted DnaJ protein contributes to maintenance of photosystem II under chilling stress
Source: J Exp Bot. 2013 Nov 13;65(1):143–58. doi: 10.1093/jxb/ert357 (PMC3883286; doi:10.1093/jxb/ert357)
Supplement: Supplementary Data [file supp_ert357_jexbot109462_file001.pdf]

A chloroplast-targeted DnaJ protein contributes to maintenance of photosystem II under chilling stress

Authors: Fanying Kong, Yongsheng Deng, Bin Zhou, Guodong Wang, Yu Wang, Qingwei Meng*

Supplement legends

Supplementary Fig. S1. Response of *LeCDJ1* to chilling stress in darkness. qRT-PCR analysis of the response of *LeCDJ1* to chilling stress in darkness. The transcript level of *LeCDJ1* was normalized to *EF-1α* expression. Error bars represent the SDs of triplicate reactions.

Supplementary Fig. S2. Amino acid sequence alignment between LeCDJ1 and DnaJ8. The accession numbers in GenBank of DnaJ8 are as follows: LeCDJ1 (GQ925907), *Lycopersicon esculentum*; AtDnaJ8 (NP_178207.1), *Arabidopsis thaliana*; PsJ8b (ADL32216), *Pisum sativum*; GmDnaJ8 (ACU18989), *Glycine max*; MtDnaJ8 (ACJ83936), *Medicago truncatula*; RcDnaJ8 (EEF49240), *Ricinus communis*; and VvDnaJ8 (XP_002263153), *Vitis vinifera*.

Supplementary Table S1. Specific primers used in this study.

Supplementary Table S2. Prediction of subcellular localization of LeCDJ1 and cpHsp70 by software program ChloroP 1.1. Gene is the name of the submitted sequence. Length is the length of the submitted sequence. Score is the output score for the probability of protein localization in the chloroplast. cTP predicts whether a sequence is a cTP-containing sequence, wherein "Y" signifies that the sequence is predicted to contain a cTP. cTP-length is the predicted length of the presequence. cTP, chloroplast transit peptide. ChloroP 1.1 (http://www.cbs.dtu.dk/services /ChloroP/).

Supplementary Table S3. Prediction of subcellular localization of LeCDJ1 and cpHsp70 using software program TargetP 1.1. Gene is the name of the submitted

sequence. Numbers given under the cTP, mTP, SP, and others represent the probabilities of protein localization in different subcellular regions (cTP, chloroplast transit peptide; mTP, mitochondrial targeting peptide; SP, secretory pathway signal peptide). TargetP 1.1 (http://www.cbs.dtu.dk/services /TargetP/).

Supplementary material

Fig. S1.

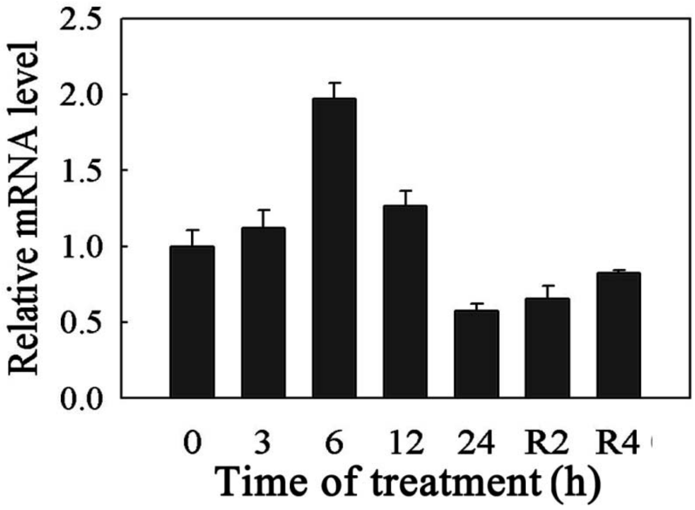

Fig. S2.

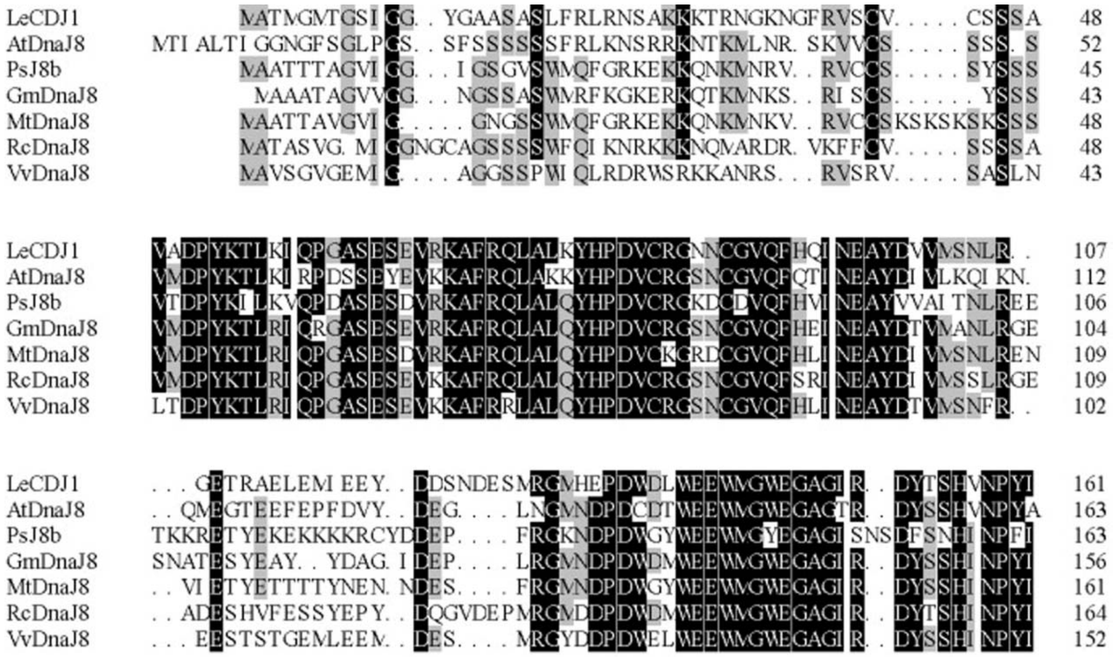

Table S1. Specific primers used in this study.

Name	*Sequence*	*Product using*
JF	CAATGGCTACGATGGGAATGA	Gene isolation and transgenic plants
JR	AGAGCAAACA AAGAGGGAATC	identification
JGF	GCAATGGCTACGATGGGAAT	Analysis of subcellular localization
JGR	GTCACGAATGCCAGCAC	
JNF	CAATGGCTACGATGGGAAT	RNA gel blot
JNR	TGCATACGTCTGGGTGATACT	
JRF	ACTCAGCGAAGAAGAAGACTAG	qRT-PCR
JRR	GGTGATACTTA AGAGCAAGCTG	
JJF	ATGGCTACGATGGGAATG	Yeast two-hybrid assays
JJR	CCCAAGTCATTAATTTAACAT	
*EF-1α*F	GGAACTTGAGAAGGAGCCTAAG	qRT-PCR
*EF-1α*R	CAACACCAACAGCAACAGTCT	
70F	AACGTCTTACTGAAGCTGCT	Yeast two-hybrid assays
70R	ATTCTTAATTGCTTTCGCTGAAGT	
35S	GACGCACAATCCCACTATCC	transgenic plants identification

Table S2. Prediction of subcellular localization of LeCDJ1 and cpHsp70 by software program ChloroP 1.1. Gene is the name of the submitted sequence. Length is the length of the submitted sequence. Score is the output score for the probability of protein localization in the chloroplast. cTP predicts whether a sequence is a cTP-containing sequence, wherein "Y" signifies that the sequence is predicted to contain a cTP. cTP-length is the predicted length of the presequence. cTP, chloroplast transit peptide. ChloroP 1.1 (http://www.cbs.dtu.dk/services /ChloroP/).

Gene	*Length*	*Score*	*cTP*	*cTP-length*
LeCDJ1	162	0.56	Y	45
cpHsp70	692	0.548	Y	69

Table S3. Prediction of subcellular localization of LeCDJ1 and cpHsp70 using software program TargetP 1.1. Gene is the name of the submitted sequence. Numbers given under the cTP, mTP, SP, and others represent the probabilities of protein localization in different subcellular regions (cTP, chloroplast transit peptide; mTP, mitochondrial targeting peptide; SP, secretory pathway signal peptide). TargetP 1.1 (http://www.cbs.dtu.dk/services /TargetP/).

Gene	*cTP*	*mTP*	*SP*	*other*
LeCDJ1	0.808	0.191	0.012	0.133
cpHsp70	0.822	0.057	0.012	0.318
